# Drinking well water and occupational exposure to Herbicides is associated with chronic kidney disease, in Padavi-Sripura, Sri Lanka

**DOI:** 10.1186/1476-069X-14-6

**Published:** 2015-01-18

**Authors:** Channa Jayasumana, Priyani Paranagama, Suneth Agampodi, Chinthaka Wijewardane, Sarath Gunatilake, Sisira Siribaddana

**Affiliations:** Department of Pharmacology, Faculty of Medicine, Rajarata University of Sri Lanka, Anuradhapura, 50008 Sri Lanka; Department of Chemistry, Faculty of Science, University of Kelaniya, Colombo, Sri Lanka; Department of Community Medicine, Faculty of Medicine, Rajarata University of Sri Lanka, Anuradhapura, Sri Lanka; Government Hospital, Padavi-Sripura, Sri Lanka; Department of Health Science, California State University Long Beach, Long Beach, CA USA; Department of Medicine, Faculty of Medicine, Rajarata University of Sri Lanka, Anuradhapura, Sri Lanka

**Keywords:** Chronic Kidney disease, Tubulointerstitial nephritis, Well water, Herbicides, Glyphosate, Sri Lanka

## Abstract

**Background:**

The chronic kidney disease of unknown etiology (CKDu) among paddy farmers in was first reported in 1994 and has now become most important public health issue in dry zone of Sri Lanka. The objective was to identify risk factors associated with the epidemic in an area with high prevalence.

**Methods:**

A case control study was carried out in Padavi-Sripura hospital in Trincomalee district. CKDu patients were defined using health ministry criteria. All confirmed cases (N = 125) fulfilling the entry criteria were recruited to the study. Control selection (N = 180) was done from people visiting the hospital for CKDu screening. Socio-demographic and data related to usage of applying pesticides and fertilizers were studied. Drinking water was also analyzed using ICP-MS and ELISA to determine the levels of metals and glyphosate.

**Results:**

Majority of patients were farmers (N = 107, 85.6%) and were educated up to ‘Ordinary Level’ (N = 92, 73.6%). We specifically analyzed for the effect modification of, farming by sex, which showed a significantly higher risk for male farmers with OR 4.69 (95% CI 1.06-20.69) in comparison to their female counterparts. In the multivariable analysis the highest risk for CKDu was observed among participants who drank well water (OR 2.52, 95% CI 1.12-5.70) and had history of drinking water from an abandoned well (OR 5.43, 95% CI 2.88-10.26) and spray glyphosate (OR 5.12, 95% CI 2.33-11.26) as a pesticide. Water analysis showed significantly higher amount of hardness, electrical conductivity and glyphosate levels in abandoned wells. In addition Ca, Mg, Ba, Sr, Fe, Ti, V and Sr were high in abandoned wells. Surface water from reservoirs in the endemic area also showed contamination with glyphosate but at a much lower level. Glyphosate was not seen in water samples in the Colombo district.

**Conclusion:**

The current study strongly favors the hypothesis that CKDu epidemic among farmers in dry zone of Sri Lanka is associated with, history of drinking water from a well that was abandoned. In addition, it is associated with spraying glyphosate and other pesticides in paddy fields. Farmers do not use personnel protective equipments and wears scanty clothing due to heat when spraying pesticides.

## Background

A chronic kidney disease (CKD) with unusual characteristics was first reported in 1994 among middle-aged paddy farmers in Padaviya farming colony in the northeastern boarder of the North Central Province (NCP) of Sri Lanka [1]. Some authors used the term CKDu to denote this condition where “u” stands for unknown or uncertain etiology. Twenty years after the first report, this disease is the most important public health issue in NCP with more than 50,000 estimated patients, and spreading on an epidemic scale to other farming areas in the Northern, Eastern, North Western, Central, and Uva provinces of the country [2]. The prevalence of the disease among those aged 15–70 years is estimated at 15.1% in the Anuradhapura district in NCP [3]. The unique feature of this CKD epidemic is that its etiology does not link to commonly known risk factors for CKD such as diabetes mellitus, hypertension, and glomerulonephritis [3].

The CKD patients in this epidemic are asymptomatic until the later stages of the disease and show tubular interstitial nephritis associated with mononuclear cell infiltration with development of glomerular sclerosis and tubular atrophy [4]. The characteristic geographical distribution of the disease and associated socioeconomic factors are suggestive of environmental and occupational origins. Tubulo-interstitial damage and negative immune-fluorescence for IgG, IgM, and complement-3 are more in favor of a toxic nephropathy [5]. Several research studies conducted to determine the cause of CKDu, have speculated about the causative role of agrochemicals. Toxins postulated so far as etiological factors include arsenic (As), cadmium (Cd), nephrotoxic pesticides and fluoride [1, 2, 6]. However, none of the studies performed so far has focused on the link between the disease and the exact type of pesticides used. The objective of this study was to identify the major risk factors associated with the CKDu in the Padavi-Sripura area in Sri Lanka.

## Methods

### Study setting

Sripura is a divisional secretariat of Trincomalee district of the Eastern province bordering to NCP with a population of 11,858. This area has been identified as having one of the highest prevalence of CKDu. Authors carried out a case control study to determine the association between CKDu and selected socio-demographic factors, and a number of variables related to the use of agrochemicals.

### Selection of cases

All patients (125) with CKDu were recruited from the patients who attended a medical clinic at Padavi-Sripura divisional hospital in Sri Lanka. Cases were defined as subjects diagnosed with CKD and defined as CKDu according to the criteria defined by the Sri Lanka Ministry of Health [7].

1. No past history of, or current treatment for, diabetes mellitus or chronic and/or severe hypertension, history of snakebite, urological disease of known etiology or glomerulonephritis.
2. Normal Glycosylated Hemoglobin level (HbA1C˂6.5%).
3. Blood Pressure ˂160/100 mmHg untreated or ˂140/90 mmHg on up to two antihypertensive agents.

The patients with CKDu were referred to the consultant nephrologist and the diagnosis was confirmed. All confirmed cases of CKDu was recruited. Data collection started in April 2012 and ended in October 2012.

### Selection of controls

Controls were selected from the same Padavi-Sripura area as the cases, and shared the same environment as the cases. Screening for controls consists of testing for serum creatinine and urine microalbumin.180 healthy individuals who came to the hospital for screening for CKDu during the study period were selected as controls after excluding CKDu and other chronic diseases. Controls were not matched for age.

Medical officers and university academics that were trained collected demographic and risk factor data using a pre-designed questionnaire. Age, sex, marital status, education, current and past sources of drinking water, history of smoking, illicit alcohol usage, chewing betel and tobacco, chronic use of painkillers-particularly Non-Steroidal Anti Inflammatory Drugs (NSAIDs), and family history of death due to CKD were collected. The participants’ place of residence and the predominant occupation during the last ten years were recorded. The information on whether the participants are presently drinking from well, tap, spring or water from reservoirs was collected. They were also asked about source of drinking water used previously (during the last ten years) and specifically probed about history of drinking water from a well that is currently abandoned. This was because water from some wells has become distasteful over time and people have stopped drinking from these wells (abandoned wells). These wells were defined as abandoned wells for the purpose of this study.

A detailed history of applying fertilizer (which is usually performed manually with bare hands) and pesticides (done with hand held sprayers) was obtained. The type of pesticide most widely used during the respondent’s entire lifetime was also recorded. The use of organophospates, paraquat, 2-methyl-4-chlorophenoxyacetic acid (MCPA), glyphosate, bispyribac, carbofuran, mancozeb and other commonly used pesticides were categorically identified. As many farmers did not recognize the type of pesticide by their chemical name a list of commonly available trade names were used to collect the data. These multiple brands were later lumped together based upon their chemical name. Ethical clearance for the study was obtained from the ethics review committee of the Faculty of Medicine, Rajarata University of Sri Lanka. Written informed consent was obtained from all participants.

### Water analysis

Water samples were collected at 34 wells in Padavi-Sripura and two reservoirs (Padaviya wewa and Jayanthi wewa). Padaviya wewa (2357 ha) was built in 5th century A.C and was renovated in 11 century A.C and in 1956. Jayanthi wewa belongs to the cascade system of Padaviya wewa [8]. Water samples were also collected from two wells and three different locations of mainline pipe born water from Colombo district, a non-endemic area for CKDu. Also a sample of water from a domestic reverse osmosis (RO) machine in Padavi-Sripura was collected. 18 of the wells tested at Padavi-Sripura were abandoned or not used for drinking. 200 mL polyethylene bottles were decontaminated by repeated soaking in 2 M nitric acid (24 h) and were used for water collection. Following the collection of samples, bottle were bagged and then stored in a cooler box. All bottles were refrigerated (under 4°C) and subsequently transported in a cooler box to the Institute for Integrated Research in Materials, Environments and Society (IIRMES) lab, California State University, Long Beach (CSULB), USA where they were analyzed. We measured mono and polyvalent cations in the water to test a hypothesis about heavy metals and elements contributing to water hardness as a possible cause in CKDu [9].

### ICP MS analysis

Major and trace elements were measured using an Inductively Coupled Plasma Mass Spectrometer (ICP-MS; HP 4500, Agilent Technologies, Palo Alto, CA) equipped with a quadrupole analyzer and octopole collision/reaction cell that can be pressurized with either a hydrogen (H_2_) or helium (He) reaction gas. Sample was injected at the rate of 0.4 mL/min using a peristaltic pump. Carrier Argon (Ar) gas at rate of 1.2 L/min through a Babbington-style nebulizer was introduced into a Peltier-cooled double-pass spray-chamber at 2°C. The 1.0 L/min auxiliary Ar and 12.0 L/min plasma gas Ar were added for a total of 14.2 L/min separated from nickel cones. The ICP-MS was tuned under standard settings by running the manufacturer's recommended tuning solution containing 10 μg/L of Li, Y, Ce, Tl, and Co (Agilent internal standard mix) for resolution and sensitivity. Interference levels were reduced by optimizing plasma conditions to produce low oxide and doubly charged ions (formation ratio of <1.0%) and residual matrix interferences were removed using the collision/reaction processes in the Octopole Reaction System.

### Accuracy, precision and detection limits

Accuracy was measured using the spiked standard solutions (Agilent Technologies). Ultrapure water (MilliQ) was used as blank (1 blank per each 10 sample batch).

Precision (reproducibility) was ascertained using within-day replicate analysis of samples. The Relative Standard Deviation (%RSD = SD/χ of the replicate values X 100%; χ is mean value) was calculated to give an indication of sample preparation and analytical precision. Replicates of each day provided an indication of within-day precision.

The analytical detection limit was calculated as the concentration of the element which gave a detectable signal above the background noise at greater than the 99% confidence level, and the detection limit was calculated as the mean of blanks plus 3 times the standard deviation of the mean.

### Detection of glyphosate

We decided to measure glyphosate because two current authors have formulated and published a detailed hypothesis that incriminates glyphosate that chelates with heavy metals as a causative factor for CKDu and also contributing to water hardness [9]. Enzyme-immunoassay in the analysis of glyphosate in water is a cost effective and reliable method. ELISA method has a lower detection limit of glyphosate (0.6 μg/L) compared to HPLC method (50 μg/L) although there was no statistically significant difference between two methods [10].

Water samples were tested for glyphosate by Enzyme-immunoassay using commercial test kits (US Biocontract Inc., San Diego, CA) according to the manufactures protocol. This test is based on the competition between the glyphosate and glyphosate-horseradish peroxidase conjugate for binding to the rabbit antibody raised against glyphosate. Validation of the ELISA test was done in comparison with GC-MS. To study the recovery rate several samples were spiked with 10 μg/L of glyphosate and it was measured in the supernatant by using ELISA.

### Data analysis

Associations were investigated with frequency tables. Continuous variables were summarized by means and standard deviations. Categorical variables were dichotomized for analyses. Odds ratios (OR), 95% confidence intervals, and p-values were obtained to assess the association between exposure and developing CKDu. Data were analyzed using SPSS (version 22).

## Results

Of the 125 diagnosed CKDu patients, significantly higher number (n = 89, 71.2%) was male. The mean age of male (45.51 ± 19.78 years) and female (47.45 ± 17.51 years) patients did not show any significant difference. Majority of the CKDu patients included in the study had education up to ordinary level (n = 92, 73.6%) and were farmers (n = 107, 85.6%). Only nine (7.2%) CKDu patients reported death of a close family member due to CKD. Of the CKDu patients, 86 (96.6%) males were farmers compared to 21 (58.3%) females. Controls included 180 healthy individual presented to the study site for screening for CKDu. This included 98 (54.4%) males and 82 (45.6%) females.

### Factors associated with CKDu

In the univariable analysis (Table 1), male sex (OR 2.07, 95% CI 1.27-3.36), engaging in farming related activities (OR 3.12, 95% CI 1.74-5.61), pesticide application (OR 3.31, 95% CI 2.04-5.36), applying fertilizers (OR 2.37, 95% CI 1.43-3.93), drinking well water (OR 4.82, 95% CI 2.27-10.24) and a history of drinking from recently abandoned well (OR 6.93, 95% CI3.87-12.40) was significantly associated with CKDu. We specifically analyzed for the effect modification of, farming by sex, which showed a significantly higher risk for male farmers with OR 4.69 (95% CI 1.06-20.69) in comparison to their female counterparts. CKDu was associated with life time exposure to different kinds of pesticides considered in this study except carbofuran. Glyphosate, a total weed killer, was the most widely used pesticide among farmers in both groups. In general, subjects who sprayed glyphosate were four times more likely to have CKDu compared to those without such a history.

**Table 1:** **The distribution the demographic variables and other risk factors, by CKDu outcome**

	CKDu Patients (Cases) N = 125(%)	Non CKD (Controls) N = 180 (%)	Unadjusted OR (95% CI) p-value
Gender			
Male	89 (71.2)	98 (54.4)	2.07 (1.27-3.36) 0.0031*
Female	36 (18.8)	82 (45.6)	
Education			
Up to OL	92 (73.6)	147 (81.7)	0.64 (0.37-1.10) 0.1041
Higher education	33 (26.4)	33 (18.3)	
Farming			
Yes	107 (85.6)	118 (65.6)	3.12 (1.74-5.61) < 0.0001*
No	18 (14.4)	62 (34.4)	
Pesticide application			
Yes	86 (68.8)	72 (40.0)	3.31 (2.04-5.36) <0.0001*
No	39 (31.2)	108 (60.0)	
Applying Fertilizer			
Yes	95 (76.0)	103 (57.2)	2.37 (1.43-3.93) < 0.0001*
No	30 (24.0)	77 (42.8)	
Drinking well water			
Yes	116 (92.8)	131 (72.8)	4.82 (2.27-10.24) < 0.0001*
No	9 (7.2)	49 (27.2)	
History of drinking water from abandoned well			
Yes	58 (46.4)	20 (11.1)	6.93 (3.87-12.40) < 0.0001*
No	67 (53.6)	160 (88.9)	
Smoking			
Yes	36 (28.8)	37 (20.6)	1.56 (0.92-2.66) 0.0970
No	89 (71.2)	143 (79.4)	
Illicit alcohol			
Yes	17 (13.6)	21 (11.7)	1.19 (0.60-2.36) 0.6150
No	108 (86.4)	159 (88.3)	
Betel chewing			
Yes	106 (84.8)	152 (84.4)	1.03 (0.55-1.94)0.9326
No	19 (15.2)	28 (15.6)	
Tobacco with betel chewing			
Yes	92 (73.6)	129 (71.7)	1.10 (0.66-1.84) 0.7101
No	33 (26.4)	51 (28.3)	
Long term pain killer use			
Yes	10 (8.0)	15 (8.3)	0.96 (0.42-2.20) 0.9169
No	115 (92.0)	165 (91.7)	
Family history of death due to CKD			
Yes	9 (7.2)	13 (7.2)	1.00 (0.41- 2.41) 0.9941
No	116 (92.8)	167 (92.8)	
Ever used Organophosphate			
Yes	54 (43.2)	54 (30.0)	1.77 (1.10-2.86) 0.0183*
No	71 (56.8)	126 (70.0)	
Ever used Paraquat			
Yes	64 (51.2)	53 (29.4)	2.51 (1.56-4.04) 0.0001*
No	61 (48.8)	127 (70.6)	
Ever used MCPA			
Yes	56 (44.8)	56 (31.1)	1.80 (1.12-2.88) 0.0152*
No	69 (55.2)	124 (68.9)	
Ever used Glyphosate			
Yes	82 (65.6)	55 (30.6)	4.33 (2.66-7.05) <0.0001*
No	43 (34.4)	125 (69.4)	
Ever used Bispyribac			
Yes	64 (51.2)	62 (34.4)	2.00 (1.25-3.18) 0.0037*
No	61 (48.8)	118 (65.6)	
Ever used Carbofuran			
Yes	46 (36.8)	51 (28.3)	1.47 (0.91-2.40) 0.1192
No	79 (63.2)	129 (71.7)	
Ever used Mancozeb			
Yes	46 (36.8)	51 (28.3)	1.94(1.21-3.13) 0.0062*
No	79 (63.2)	129 (71.7)	

*P < 0.05.

In the multivariable analysis, we investigated the un-confounded effect of individual exposure factors after adjustment for age, sex, educational level, having a death among family members due to CKD and other exposure factors related to agrochemical use. Drinking well water (OR 2.52, 95% CI 1.12-5.70), history of drinking from recently abandoned well (OR 5.43, 95% CI 2.88-10.26) and use of glyphosate 5.12 (2.33-11.26) were the factors significantly associated with CKDu. A significant difference between groups has been detected with related to pesticide application (OR 2.34: 95% CI 0.97- 5.57) however, perhaps with a smaller sample size, this difference would have proved to be not statistically significant.

### Analysis of drinking water samples

There was a significant difference between abandoned (not used for drinking now) and serving wells (presently used for drinking) in total permanent hardness (calculated value using calcium and magnesium). Barium, iron and strontium were significantly high in abandoned wells (Table 2).

**Table 2:** **Hardness and related parameters in drinking water samples**

	mg/L				μg/L		mg/L	μs/cm
Well type	Na	K	Ca	Mg	Ba	Sr	Hardness	Condutivity
Abandoned wells in Sripura (n = 18)								
Mean	74.8	0.9	51.8	28.0	150.7	572.4	243.6	877.2
Median	57.1	0.8	53.2	29.6	142.3	519.0	234.5	623.0
Serving wells in Sripura (n = 16)								
Mean	51.9	0.6	29.0	13.0	89.4	269.1	125.9	465.0
Median	38.6	0.7	28.5	11.6	83.9	265.0	121.0	509.5
U	77.5	97.0	26.5	24.5	64.0	31.0	6.0	44.0
Z^#^	−2.295	−1.629	−4.054	−4.124	−2.760	−3.899	−4.762	−3.450
P Value	.0217*	.1034	.0001**	.0000**	0.0058**	.0001**	.0000**	.0006**
Surface water	5.85	0.45	10.6	1.15	10.4	46	31.2	79.5
RO water	3.1	0.2	0.6	0.6	2.1	4.6	4.0	22.0
Colombo well	2.6	0.7	6.8	0.8	16.4	13.1	20.2	49
Colombo main	2.5	0.7	6.8	0.8	16.5	14.5	20.0	49

*P < 0.05, **P < 0.01, # two sample Wilcoxan rank-sum (Mann–Whitney) test.

According to the classification by United States Geological Survey on water hardness [11], all the other abandoned wells were classified as having very high hardness except for two. However, none of the currently serving wells can be classified as such and belongs to either moderately hard or hard category of water hardness. Surface water from Padaviya reservoir had very low hardness (i.e. soft) that was similar to the well water and mainline pipe borne water in the Colombo district. In parallel, calcium, magnesium, barium and strontium levels for water in non-endemic Colombo district was also low in comparison to the endemic area.

High conductance indicates high concentration of dissolved-solids. This can affect the palatability of water. Conductivity was highest among the abandoned wells. Content of iron in abandoned wells was also significantly high.

Glyphosate (Gly) concentration also showed a marked difference between serving and abandoned wells (Table 3). All the abandoned wells except for one (94%) contained more than 1 μg/L of glyphosate where as among the serving wells only 31% contained glyphosate above the 1 μg/L level. There was trace amounts of glyphosate in the surface water in two reservoirs indicate the glyphosate may have been washed off from the agricultural or paddy lands. However glyphosate was not seen in well or mainline pipe borne water in Colombo district.

**Table 3:** **Trace metal and glyphosate content in drinking water samples**

	Abandoned wells		Serving wells		U	Z ^#^	P value	Surface water	Ro water	Colombo well	Colombo tap
	Mean	Median	Mean	Median							
Al	2.2	1.0	2.9	2.2	117.5	−0.916	0.3596	3.5	<0.1	6.7	71.6
Sb	3.2	0.9	4.5	2.9	127.0	−0.587	0.5570	3.4	0.1	0.3	0.5
As	0.8	0.4	0.3	0.3	104.5	−1.381	0.1674	0.2	0.2	<0.5	<0.1
Cd	0.04	0.0	0.04	0.0	138.0	−0.247	0.8051	0.1	0.1	0.1	0.1
Cr	1.0	0.6	0.5	0.0	103.0	−1.495	0.1348	0.3	0.4	<0.1	<0.1
Co	0.07	0.1	0.2	0.1	118.0	−0.968	0.3329	0.1	<0.1	0.1	0.2
Cu	1.4	1.2	1.9	1.0	107.5	−1.264	0.2063	0.4	3.5	0.3	0.4
Fe	90.5	76.6	45.2	40	60.0	−2.898	0.0038**	20.0	1.5	17.4	26.1
Pb	0.5	0.1	0.2	0.1	124.0	−0.791	0.4287	0.1	0.2	0.1	0.1
Mn	2.8	1.1	2.0	0.3	98.5	−1.573	0.1156	0.8	0.4	5.5	6.4
Mo	1.5	1.4	1.1	0.8	97.5	−1.609	0.1077	0.3	1.0	0.3	0.4
Ni	0.8	0.7	3.1	0.6	140.0	−0.139	0.8897	0.3	0.8	0.1	0.2
Se	0.7	0.7	0.3	0.2	65.5	−2.731	0.0063**	0.4	0.3	<0.1	<0.1
Ag	0.01	0.0	0.02	0.0	133.5	−0.736	0.4615	<0.1	0.1	<0.1	0.1
Tl	0.1	0.1	0.05	0.0	129.0	−0.582	0.5603	0.1	0.1	<0.1	<0.1
Sn	0.3	0.3	0.3	0.3	143.0	−0.038	0.9696	0.3	0.2	0.3	0.3
Ti	0.8	0.8	0.6	0.7	85.5	−2.047	0.0406*	0.2	0.3	0.3	0.3
V	14.4	10.5	17.4	6.3	84.5	−2.053	0.0400*	1.4	0.8	0.3	0.3
Zn	4.3	2.3	7.7	3.4	115.0	−1.001	0.3168	6.8	6.1	0.8	0.7
Gly^+^	3.5	3.2	0.7	0.6	15.0	−4.456	0.0000**	0.05	ND	ND	ND

*P < 0.05, **P < 0.01, # two sample Wilcoxan rank-sum (Mann–Whitney) test, ^+^ Glyphosate.

## Discussion

The present study revealed that male farmers from Padavi-Sripura, who spray glyphosate, drink well water and had history of drinking from an abandoned well, are at a significantly higher risk of developing CKDu. This association is evident even after adjusting for all the baseline and exposure variables. This is the first study in Sri Lanka that analyses the association of CKDu among farmers with the type of pesticide and most widely used pesticide during their lifetime.

The only pesticide not associated with CKDu in univariable analysis is carbofuran an insecticide in granular form. All other pesticides considered in this study are in liquid form and easily absorbable through skin and by inhalation, particularly during manual spray. Paraquat was a common herbicide used by the paddy farmers since 1960s [12]. However, it was phased out gradually due to 400 to 500 deaths per year in Sri Lanka due to its use for committing suicide [13]. Paraquat also can cause respiratory, skin and mucosal irritation among those who spray it [14]. In 2010 pesticide technical advisory committee of Sri Lanka decided to ban the use of paraquat for paddy cultivation [15].

The ban on paraquat prompted most farmers to switch to using glyphosate as an alternative herbicide. There are no immediate symptoms among sprayers of glyphosate. However, there are important differences between these two pesticides. Paraquat is a contact herbicide but glyphosate is a trans-locating herbicide with anti-microbial action that softens the earth, paving the way for ‘till-less’ farming [16]. Glyphosate came to the market in 1971 but there are no details of when it was first available in Sri Lanka. However, it has become most widely used pesticide in Sri Lanka [9]. The amount of glyphosate imported was 5.3 million kg and this represents more than half (52%) of the total pesticides (including all other herbicides, insecticides, and fungicides) imported in 2012 [9]. There are no studies that report nephrotoxic properties of glyphosate on humans. However, several animal studies provide evidence for nephrotoxic properties of glyphosate [17–22]. Toxicity of glyphosate to common hourglass tree frog in Sri Lanka is previously reported [23].

Byspiribac which is another weed killer also increases the risk of CKDu. Organophosphate compounds and moncozeb, are the leading insecticide and fungicide respectively, used by the farmers in the Padavi-Sripura area. The use of all these pesticides increases the risk of getting CKDu with people who used glyphosate having the highest risk.

Certain pesticides are known to be associated with CKDu in humans. A study done in India demonstrated that there was a significant correlation between plasma levels of organochlorine and decrease in GFR and the development of CKD [24]. Internationally, the use of organochlorine and arsenical pesticides at cotton fields in the Aral Sea area which caused an epidemic of tubular kidney damage and abnormal increase of non-communicable diseases in central-Asia, is considered to be one of the world’s greatest ecological disasters [25–27]. Moreover, a CKDu epidemic very similar to that of Sri Lanka has been identified in agricultural communities in Andra and Odisha, two southeastern provinces of India [28, 29], upper Egypt [30] and predominantly among young male farm workers in the pacific coastal region of Central American countries of El Salvador, Nicaragua, and Costa Rica [31, 32]. Similar to Sri Lankan and Indian scenarios the etiology of CKDu in Mesoamerica is not linked to the most frequently known causes such as diabetes mellitus and hypertension.

Drinking water previously from an abandoned well increases the risk of developing CKDu almost seven times in univariable analysis. Drinking well water and having a history of drinking water from a well abandoned within the last 10 years are significant predictors of CKDu. The abandoning of certain shallow wells in which water has previously been used for drinking purposes is a common phenomenon in the CKDu endemic area. Long-term residents in this area complain that the ground water hardness has been steadily increasing over the last two decades rendering the water increasingly unpalatable and not suitable for cooking.

Hardness of water from abandoned wells is associated with the presence of metals such as calcium and magnesium. Aluminum, barium, iron, manganese, strontium and zinc also contribute to the hardness in water but not shown here in the calculation of traditional hardness [33]. These metals contribute to the increased dissolved solids and enhanced conductivity of the water. Unpalatability and the inability to use it for cooking purposes may have driven people to abandon these wells. These conditions have made it necessary for the residents in the endemic area to travel longer distances to obtain water from the currently serving wells, which are becoming lesser in numbers as more and more wells get abandoned due to increasing hardness. Most of the residents indicate that the hardness and the objectionable taste are on the increase even in the currently serving wells in comparison to the surface water. The poor taste and the scarcity of the water itself may cause people in the Padavi-Sripura to drink less water thus contributing to further dehydration and deteriorating renal function. Organic compounds in the water also can contribute towards the taste of water. The unknown factor here is glyphosate. Do the pesticides contribute to the water taste? Another aspect of these herbicides (glyphosate, bispyribac, MCPA, and paraquat) is that they process one or more carboxyl groups that are capable of retaining metals [34–36]. Calcium, Magnesium and Strontium ions present in hard water and heavy metals present in agrochemicals combine with pesticide structures and resist quick degradation within the environment [9]. This may be a reason for the gradual increase in hardness and objectionable taste in the water of abandoned shallow wells in the endemic areas.

If drinking the water contaminated with heavy metals and glyphosate, from abandoned wells in the past, and now from the currently serving wells, is the only risk factor in operation, then, we can expect to see all inhabitants in the endemic area- males and females, farmers and non-farmers- to be equally affected by the CKDu. As this is not the case, we now have to provide an explanation as to why the male sex, farming and applying pesticides increase the risk of developing CKDu by almost three fold. The most plausible explanation here is that the risk of CKDu associated with drinking water contaminated with heavy metals and pesticides represent only the important baseline risk, for both males and females. However, the continued exposure to glyphosate and other pesticides through manual spraying and other farming activities can augment this baseline risk several fold, particularly for the male farmers. Due to the strenuous exertion needed for carrying a 16 L or 20 L metal sprayer full of liquid pesticides on their back for several hours, the spraying function has been exclusively delegated to the male farmers. This type of manual spraying of glyphosate can expose the male farmers to the pesticide chronically. The presence of significant levels of glyphosate in the urine of the farmers and their family members several hours after spraying the pesticide has been well documented [37, 38]. In one study the authors attribute these high levels of glyphosate to the oral and dermal absorption during and immediately after spraying [38]. Acquavella et al. have demonstrated that the farmers who do not use Personal Protective Equipment (PPE) such as gloves, have five times more glyphosate levels in their urine in comparison to those who use PPE properly [37]. In a focus group discussion conducted by two current authors (SG and CW) 16 farmers from Padavi-Sripura area, admitted that they use very little or no PPE in spraying pesticides and wear only scanty clothing during the process, primarily because of the unbearable heat in the paddy fields. These farmers also admitted that many of their colleagues do not follow instructions and the specified guidelines for spraying and often use much higher concentrations of the pesticide in the hope of obtaining better results. All of the facts stated above can now account for the significantly high risk and the associations observed among the male farmers who spray glyphosate.

The mean age of the male patients with CKDu was 45.5 years while its 47.4 years in females i.e., it is a disease of middle- farmers. However, the mean age of the both male and female patients were considerably lower when compared to those of a previous study done in 2007 [39]. In that study, the mean age of the male and female patients with CKDu was 56.7 years and 54.2 years, respectively. A decreasing mean age of patients compared to a study done 6 years ago supports the notion of the progressive nature of the disease and the possible contribution of a cumulative toxin in the pathogenesis of the disease process.

Smoking, consuming alcohol, chewing betel with or without tobacco, long-term pain killer (NSAIDs) use and family history of death due to CKD were not risk factors for developing CKDu in Padavi-Sripura region even in the univariable analysis. Level of education and illicit alcohol consumption were not significant covariates associated with increased risk of CKDu in Padavi-Sripura. Smoking and consumption of alcohol by females is culturally frowned upon in Sri Lankan society-a phenomenon that accounts for the results seen this study.

A published study in 2010 demonstrated that, chemical fertilizers used in Sri Lanka are contaminated with cadmium, chromium, uranium and radioactive substances [40]. The possibility of cutaneous and respiratory absorption of pesticides while working in the field and long term low level exposure to arsenic, heavy metals, and pesticide residues through drinking water, food, and tobacco have already been documented [2, 37]. A study carried out in 12 countries demonstrated significant amount of cadmium in rice cultivated in Sri Lanka [41]. Tobacco that is chewed with betel by rural farmers in Sri Lanka also contains cadmium and other nephrotoxic metals [2].

The recall bias associated with this case control study, and the selection of controls from community members who came to the hospital for screening can be a drawback. However, they are from the same community with the same health anxiety about the CKDu. Ideal controls should be based within the community. We have also not matched the cases and controls. However, we have used stratification in analysis. The selection of cases also was not ideal. They were not newly diagnosed or incident cases. However, they were diagnosed between 2009 December and April 2012. A patient diagnosed in December 2009 if he or she survives, will be recruited to the study within 30 months of diagnosis. The CKDu patients have a high mortality and we have studied only survivors with different severity, different prognosis and different stages of the disease.

The in-depth details of lifetime exposure to pesticides were not gathered. As rice farming is seasonal, the number of days per year and years of use was not collected. Intensity level of pesticide exposure was also not measured along with other details such as the application method, mixing status, equipment and the use of PPE.

## Conclusions

The current study strongly supports the hypothesis that CKDu in Sri Lanka is a drinking-water-related disease in farmers who have a history of spraying glyphosate. Further studies should focus the abandoned drinking water sources in areas with high prevalence of the disease and investigate the link between CKDu and glyphosate in particular and heavy metals in drinking water.

## Authors’ information

CJ is a lecturer in Pharmacology at Faculty of Medicine, Rajarata University of Sri Lanka (RUSL). He is the Director, National project for the prevention of kidney diseases, Sri Lanka. PP is the chair and professor of Chemistry in the Department of Chemistry, University of Kelaniya, Sri Lanka. SA is the head and Senior Lecturer of Community Medicine in Faculty of Medicine, RUSL. CW was the Medical officer in charge, Padavi-Sripura government hospital. SG is Professor, California State University, Long Beach, USA and a diplomate American board of preventive medicine in occupational medicine. SS is chair and Professor of Medicine, Faculty of Medicine, RUSL.

## Abbreviations

CKDu: Chronic Kidney Disease of unknown etiology
NCP: North Central Province
NSAIDs: Non-Steroidal Anti Inflammatory Drugs
MCPA-2: methyl-4-chlorophenoxyacetic acid
IIRMES: Integrated Research in Materials, Environments and Society
CSULB: California State University, Long Beach
ICP-MS: Inductively Coupled Plasma Mass Spectrometer
SRMs: Standard reference materials
ELISA: Enzyme link immune sorbent assay
HPLC: High performance liquid chromatography
PPE: Personal protective equipment.

## References

[1] Jayasumana MACS, Paranagama PA, Amarasinghe MD, Wijewardane KMRC, Dahanayake KS, Fonseka SI (2013). Possible link of Chronic arsenic toxicity with Chronic Kidney Disease of unknown etiology in Sri Lanka. J Nat Sci Res.

[2] Ministry of Health (2014). Data Presented at the presidential task force for prevention of kidney diseases.

[3] Jayatilake N, Mendis S, Maheepala P, Mehta FR (2013). Chronic kidney disease of uncertain aetiology: prevalence and causative factors in a developing country. BMC Nephrol.

[4] Nanayakkara S, Komiya T, Ratnatunga N, Senevirathna STMLD, Harada KH, Hitomi T (2012). Tubulointerstitial damage as the major pathological lesion in endemic chronic kidney disease among farmers in North Central Province of Sri Lanka. Environ Health Prev Med.

[5] Athuraliya NT, Abeysekera TD, Amerasinghe PH, Kumarasiri R, Bandara P, Karunaratne U (2010). Uncertain etiologies of proteinuric-chronic kidney disease in rural Sri Lanka. Kidney Int.

[6] Wanigasuriya K (2012). Aetiological factors of Chronic Kidney Disease in the North Central Province of Sri Lanka: A review of evidence to-date. J Coll Comm Physic Sri Lanka.

[7] Secretary to the Ministry of Health (2009). Chronic Kidney Disease of Unknown Etiology; Circular no 01-10/2009.

[8] Jayasena HAH, Chandrajith R, Gangadhara KR (2011). Water management in ancient Tank Cascade Systems (TCS) in Sri Lanka: Evidence for systematic tank distribution. J Geol Soc Sri Lanka.

[9] Jayasumana C, Gunatilake S, Senanayake P (2014). Glyphosate, hard water and nephrotoxic metals: Are they the culprits behind the epidemic of chronic kidney disease of unknown etiology in Sri Lanka?. Int J Environ Res Public Health.

[10] Rubio F, Veldhuis LJ, Clegg BS, Fleeker JR, Hall JC (2003). Comparison of a direct ELISA and an HPLC method for glyphosate determinations in water. J Agric Food Chem.

[11] **Water Hardness Classification of the United States Geological Survey** [https://water.usgs.gov/owq/hardness-alkalinity.html]

[12] Wilks MF, Fernando R, Ariyananda PL, Eddleston M, Berry DJ, Tomenson JA (2008). Improvement in survival after paraquat ingestion following introduction of a new formulation in Sri Lanka. PLoS Med.

[13] Dawson A, Buckley N (2007). Integrating approaches to paraquat poisoning. Ceylon Med J.

[14] Castro-Gutiérrez N, McConnell R, Andersson K, Pacheco-Antón F, Hogstedt C (1997). Respiratory symptoms, spirometry and chronic occupational paraquat exposure. Scand J Work Environ Heal.

[15] Gawarammana IB, Buckley NA (2011). Medical management of paraquat ingestion. Br J Clin Pharmacol.

[16] Szekacs A, Darvas B, Hasaneen MN (2012). Forty years with glyphosate. Herbicides–Properties, Synthesis and Control of Weeds.

[17] Jiraungkoorskul W, Upatham ES, Kruatrachue MSahaphong S, Vichasri-Grams S, Pokethitiyook P (2003). Biochemical and histopathological effects of glyphosate herbicide on nile tilapia (Oreochromis niloticus). Environ Toxicol.

[18] Ayoola SO (2008). Histopathological effect of glyphosate on Juvenile African Catfish(Clariasgariepinus). Am Eurasian J Agric Environ Sci.

[19] Séralini GE, Cellier D, De Vendomois JS (2007). New analysis of a rat feeding study with a genetically modified maize reveals signs of hepatorenal toxicity. Arch Environ Contam Toxicol.

[20] Tizhe EV, Ibrahim ND, Fatihu MY, Igbokwe IO, George BD (2013). Serum biochemical assessment of hepatic and renal functions of rats during oral exposure to glyphosate with zinc. Comp Clin Path.

[21] Larsen K, Najle R, Lifschitz A, Virkel G (2012). Effects of sub-lethal exposure of rats to the herbicide glyphosate in drinking water: Glutathione transferase enzyme activities, levels of reduced glutathione and lipid peroxidation in liver, kidneys and small intestine. Environ Toxicol Pharmacol.

[22] Krüger M, Schrödl W, Neuhaus J, Shehata AA (2013). Field Investigations of Glyphosate in Urine of Danish Dairy Cows. J Environ Anal Toxicol.

[23] Jayawardena UA, Rajakaruna RS, Navaratne AN, Amerasinghe PH (2010). Toxicity of Agrochemicals to Common Hourglass Tree Frog (Polypedates Cruciger) in Acute and Chronic Exposure. Int J Agri Bio.

[24] Siddharth M, Datta SK, Bansal S, Mustafa M, Banerjee BD, Kalra OP (2012). Study on organochlorine pesticide levels in chronic kidney disease patients: association with estimated glomerular filtration rate and oxidative stress. J Biochem Mol Toxicol.

[25] Kaneko K, Chiba M, Hashizume M, Kunii O, Sasaki S, Shimoda T (2003). Renal tubular dysfunction in children living in the Aral Sea Region. Arch Dis Child.

[26] Whish-Wilson P (2002). The Aral Sea Environmental Health Crisis. J Rurasl Remote Environ Heal.

[27] Kumar RS (2002). Aral Sea: Tragedy Environmental in Central Asia. Econ Polit Wkly.

[28] Reddy DV, Gunasekar A (2013). Chronic kidney disease in two coastal districts of Andhra Pradesh, India: Role of drinking water. Environ Geochem Health.

[29] **Kidney conundrum** [http://www.downtoearth.org.in/content/kidney-conundrum]

[30] El Minshawy O (2011). End-stage renal disease in the El-Minia Governorate, upper Egypt: An epidemiological study. Saudi J Kidney Dis Transpl.

[31] Orantes CM, Herrera R, Almaguer M, Brizuela EG, Hernández CE, Bayarre H (2011). Chronic kidney disease and associated risk factors in the Bajo Lempa region of El Salvador: Nefrolempa study, 2009. MEDICC Rev.

[32] Correa-Rotter R, Wesseling C, Johnson RJ (2014). CKD of unknown origin in Central America: The case for a mesoamerican nephropathy. Am J Kidney Dis.

[33] Cotruvo J (2011). Hardness in Drinking Water, Background Document for Development of WHO Guidelines for Drinking Water Quality.

[34] Nalewaja J (1991). Salt antagonism of glyphosate. Weed Sci.

[35] Nalewaja J (1993). Spray carrier salts affect herbicide toxicity to Kochia (*Kochiascoparia*). Weed Technol.

[36] Sumbramaniam V, Hoggard PE (1998). Metal complexes of glyphosate. J Agric Food Chem.

[37] Acquavella JF, Alexander BH, Mandel JS, Gustin C, Baker B, Chapman P (2004). Glyphosate biomonitoring for farmers and their families: Results from the farm family exposure study. Environ Health Perspect.

[38] Mesnage R, Moesch C, Grand R, Lauthier G, Vendômois J, Gress S (2012). Glyphosate Exposure in a Farmer’s Family. J of Env Protection.

[39] Wanigasuriya KP, Peiris-John RJ, Wickremasinghe R, Hittarage A (2007). Chronic renal failure in North Central Province of Sri Lanka: an environmentally induced disease. Trans R Soc Trop Med Hyg.

[40] Chandrajith R, Seneviratna S, Wickramaarachchi K, Attanayake T, Aturaliya TNC, Dissanayake CB (2009). Natural radionuclides and trace elements in rice field soils in relation to fertilizer application: study of a chronic kidney disease area in Sri Lanka. Environ Earth Sci.

[41] Meharg AA, Norton G, Deacon C, Williams P, Adomako EE, Price A (2013). Variation in rice cadmium related to human exposure. Environ Sci Technol.

